# Infection with an Organism of the Actinomyces Group

**Published:** 1907-10

**Authors:** Frederick J. Parmenter

**Affiliations:** Buffalo, N. Y.


### Infection with an Organism of the Actinomyces Group. Recovery.

By FREDERICK J. PARMENTER, M. D., Buffalo, N. Y.

THE patient was a male, aged 20, by occupation a dry-goods clerk; the family and personal history are unimportant. Upon returning from a two weeks' vacation in the country, during which time he overexercised and overate to great excess, he was taken suddenly ill May 29, 1905, with acute appendicitis. Immediate operation was advised and promptly carried out. The appendix had perforated and lay in an abscess cavity; it was removed, the cavity disinfected, and the wound closed without drainage. The writer was an interne in the hospital at this time and assisted at the operation. The boy made a prompt recovery and returned home about eighteen days later.

He remained well until Sept. 1, 1906, although he noticed that during the latter part of August he had to urinate quite frequently. On September 1, following another vacation in the country similar to that of 1905, he was taken with malaise, chills, and diarrhea. He partially recovered in three or four days, returned to work and remained fairly well until the tenth when a recurrence of the trouble again confined him to his bed. The writer was called to see him September 21, about 9 p.m., one year and four months after the operation for appendicitis. Examination disclosed a well developed but poorly nourished and quite anemic young man; temperature 103°F., pulse 100, rather tense and small in volume. Facial expression good, thorax negative. The abdomen showed a mass filling the lower right quadrant, extending from the right side to just beyond the median line, and over the lower two-thirds of the region between Poupart's ligament and the umbilicus. The mass was tender on palpation, dull on percussion, and the right rectus muscle was thickened and felt edematous, being in a moderate state of spasm. The diagnosis of cecal abscess of unknown origin was made from the history of acute onset, the sudden development of the mass associated with temperature and rising pulse, the patient feeling sure that the mass had developed within two weeks.

Operation was advised, consented to, and performed September 22, 1906. Incision was made through the old scar, the right rectus muscle being markedly thickened and edematous. Upon opening the peritoneum a mass was found consisting of the cecum, together with several loops of ileum and mesentery occuyping the entire lower right quadrant, extending just beyond the left lateral surface of the bladder and upward half way to the umbilicus. It was very firmly attached to all structures,—anteriorly, posteriorly, and laterally,—so firmly in fact that it was impossible to separate any part of it. A fine aspirating needle was introduced in several directions in the hope of finding a deep seated abscess, with no result. The absence of inflammatory exudate on the peritoneum rather ruled out the possibility of abscess and the failure to find any enlarged lymphatics seemed to negative the diagnosis of carcinoma.

A provisional diagnosis of sarcoma was made with infiltration of the right rectus muscle through the adhesions to the anterior abdominal wall. Tuberculosis and syphilis suggested themselves but were ruled out from lack of evidence. A cigarette drain was placed at the lower end of the wound in the forlorn hope that it might by chance after all be inflammatory and drainage become established along the puncture lines; or, if infection took place from the introduction of the needle, an avenue of escape would be afforded. Some serous discharge appeared from the drain for several days. The drain was removed on the fourth day; the temperature ranged from 100° to 101° F., and the patient's general condition remained at a standstill. About the fifth or sixth day reddening and induration of the skin were seen around the wound, together with the formation of blebs which ruptured and discharged a serous exudate.

The temperature began to go higher, ranging from 101° to 103° F., there was marked loss of weight, and increasing anemia. It seemed at this time his death must occur shortly, therefore, at his request, he was allowed to get up and go home at the end of two weeks. His weight by this time had fallen from 145 to 128 pounds, and blood examination showed hemoglobin 35%, erythrocytes 1,500,000 per cu. mm. As he lived near by he came frequently to the office to have the wound dressed. By this time granulations had formed making an area nearly the size of a hand. One day Dr. John Parmenter suggested removing some of the granulations for examination. This was about a week after he left the hospital, and three weeks from the date of operation. The specimen was sent to the pathological laboratory of the University of Buffalo, and examination was made by Dr. Charles A. Bentz.

Examination of the sections of the granulations did not support the diagnosis of sarcoma but revealed the presence of masses of ray fungi, staining fairly well with hematoxylin and strongly by Gram's method; the filaments branched freely but did not show clubbed ends in section. In teased fresh material clubbed ends were seen rarely and with difficulty, in fact their presence at all was somewhat doubtful. According to Wright (*Journal of Medical Research, May,* 1905) in rapidly spreading cases of actinomycosis little or no club formation may occur. (See Wright's Case 1, and remarks on p. 397.)

The masses of fungi were immediately surrounded by a zone of polynuclear leukocytes. The remainder of the sections gave the histology of granulation tissue, containing a few giant cells; plasma and mast cells were not seen. Both aërobic and anaërobic cultures were made but the only organism that grew was a coccus. The exact biological position of the fungus in this case must therefore remain in doubt, as it is probable that there are several varieties of ray fungi more or less closely related.

The patient's appendix had fortunately been preserved in alcohol and was secured for examination. A large number of sections from various points in the appendix were examined but no organism resembling actinomyces could be demonstrated.

The treatment instituted was both local and general. It was decided that the patient would be served best by acting as though the disease were actinomycosis although the position of the organism found in his case was somewhat doubtful. Furthermore, his death seemed inevitable unless some radical change occurred soon. Internally the patient was given potassium iodide, beginning with 15 grains before meals and gradually increased to 90, making in all 270 grains per day. After meals he was given a quarter of a grain of copper sulphate which was slowly increased to a third of a grain, making in all one grain per day. Locally the granulations were removed by the actual cautery. On alternate days there were injected deeply into the mass a 1% solution of copper sulphate on one day and iodine solution on the next, of about the same strength. The copper sulphate irritated the stomach considerably in the beginning but the patient being very persistent gradually overcame the difficulty; for in case he vomited the first capsule he immediately took another.

Local treatments, especially the injections, were very painful although morphine was frequently given before. In addition, an iron tonic was prescribed, together with much time spent in the open air, and plenty of nourishing, wholesome food. As a result of the treatment local and general improvement were noticed at once, so that the end of each week showed a definite gain both in blood and weight. From this time on his convalescence was uneventful. For purposes of comparison an examination, February 1, 1907, showed the following: Patient well developed and well nourished, weight 168 pounds., (15 pounds more than he ever weighed before), blood count normal. The mass and all induration have completely disappeared, leaving nothing but the scar of incision. He is able to work and according to his own statement never felt better or stronger in his life. At the present time, August 1, 1907, (ten and a half months after the operation), he continues to enjoy the best of health and has taken no medicine since April 1, 1907.

*General discussion.* Several questions of interest present themselves. In the first place, how did the infection occur and had it any relation with the attack of appendicitis about a year before? Three ways of infection may be considered. First, from an infected animal, which is not impossible in this case, for the patient faintly remembers that one of the cattle at the farm had some disease or other, but further than this he knows nothing. Second, through chewing infected pieces of straw, grass, or eating fruit which had been lying on the ground. Third, Wright, in the *Journal of Medical Research,* May, 1905, states his belief that it is probable that actinomyces bovis in the form of fragmented filaments may be a normal inhabitant of the mouth and gastro-intestinal tract, and that a foreign body may abrade and irritate the mucous membrane thus opening up a path for infection.[1] Although the attack of appendicitis which preceded the appearance of the later infection, naturally suggests that some relation between the two may have existed, the writer has given all the evidence in his possession. It has already been stated that the exact position of the organism found in this case was not determined, but that the treatment was carried out on the assumption that the disease was probably actinomycosis. The question, which drug cured the patient is of interest. Unfortunately we do not know, for the patient's condition was so desperate it did not seem justifiable to try first one and then the other.

Bevan in the *Journal of the American Medical Association* November 11, 1905, reports several cases, in which the lesion was extensive, where the iodine salts and the *x*-ray failed to yield results, upon the addition of copper sulphate both locally and internally, marked improvement resulted. The suggestion is also made that possibly mixed treatment in certain cases may be more beneficial than either of the salts when administered alone; the treatment of actinomycosis in this respect resembling that of syphilis, for it has been found in the latter condition mixed treatment is often the best. Local as well as constitutional administration of these salts seems to have been of distinct value in the present case.

In conclusion I desire to express my thanks to Dr. Roswell Park for suggestions in treatment, and to Drs. Charles A. Bentz

1. In connection with Wright's View the following case is of interest. A tonsil was removed in the Dispensary of the University of Buffalo by Dr. H. J. Mulford, and was sent to the laboratory to be used for teaching purposes on the supposition that it was an ordinary hypertrophied tonsil. When sections were made a cavity was found in the interior which was lined by stratified squamous epithelium, and which contained large masses of Gram positive ray fungi, with clubbed end. The masses were surrounded by numerous polynuclear leukocytes. The epithelial lining of the cavity was apparently deficient over a small area close to the mass of fungus, or the epithelial layers were thinned out and infiltrated with polynuclear lymphocytes. A few large multinuclear cells were seen. On the whole, the neighboring tissue of the tonsil did not show greatly more evidence of inflammation than is common in inflammatory hypertrophy. Karyokinetic figures were abundant in the epithelium and in the germinal centers of the lymphoid tissue. (Notes of Pathological Laboratory, University of Buffalo.)

and Herbert U. Williams for their report on the pathological findings, and helpful suggestions in the preparation of this paper.

519 FRANKLIN STREET.

